# Supplementary material for: Crawling and Gliding: A Computational Model for Shape-Driven Cell Migration
Source: PLoS Comput Biol. 2015 Oct 21;11(10):e1004280. doi: 10.1371/journal.pcbi.1004280 (PMC4619082; doi:10.1371/journal.pcbi.1004280)
Supplement: S1 Code — (ZIP) [file pcbi.1004280.s012.zip › release/tst/doc/html/globals.html]

Tissue Simulation Toolkit: File Members


Tissue Simulation Toolkit
 0.1.4.1


- Main Page
- Namespaces
- Classes
- Files

- File List
- File Members

- All
- Functions
- Variables
- Typedefs
- Macros

- a
- b
- c
- d
- e
- f
- g
- h
- i
- k
- l
- m
- n
- o
- p
- q
- r
- s
- t
- u
- v
- w
- x
- y
- z

Here is a list of all file members with links to the files they belong to:

### - a -

- AskSeed()
  : random.cpp
  , random.h

### - b -

- bgetpar()
  : parse.cpp
  , parse.h
- BLACK
  : sticky.h
- BLUE
  : sticky.h
- BOLTZMANN
  : sticky.h
- bool\_str()
  : parse.cpp
  , parse.h

### - c -

- CanWeWriteP()
  : output.h
- CFILE
  : x11graph.h
- chainHull\_2D()
  : hull.h
  , hull.cpp
- CheckFile()
  : output.cpp
  , output.h
- Chext()
  : output.h
  , output.cpp
- COMMUNICATION
  : sticky.h
- conrec()
  : conrec.cpp
  , conrec.h
- Coordinate
  : x11graph.h
- copyprob
  : ca.cpp
- Crash()
  : crash.h
  , crash.cpp

### - d -

- dgetparlist()
  : parse.cpp
  , parse.h
- DIV
  : sticky.h
- DMUT
  : sticky.h
- DSQR
  : sqr.h

### - e -

- EMPTY
  : sticky.h
- ENDOFSTATES
  : sticky.h
- ENERGYOFFSET
  : sticky.h
- errno
  : x11graph.cpp
- error()
  : warning.cpp
  , warning.h
- EXTERNAL\_OFF
  : dish.cpp

### - f -

- FAC
  : random.h
- FALSE
  : misc.h
  , sticky.h
  , output.h
- fgetpar()
  : parse.h
  , parse.cpp
  , parse.h
- FileExists()
  : misc.cpp
  , misc.h
- FileExistsP()
  : output.h
  , output.cpp
- FNAMESIZE
  : output.cpp

### - g -

- GetFileName()
  : misc.cpp
  , misc.h
- GREEN
  : sticky.h
- GRIDX
  : sticky.h
- GRIDY
  : sticky.h

### - h -

- HandleSIGINT()
  : crash.cpp
  , crash.h
- HandleSIGSEGV()
  : crash.h
  , crash.cpp
- HASHCOLNUM
  : cell.cpp
- HASHCOLPRIME
  : sticky.h
- HOSTDEAD
  : sticky.h

### - i -

- igetpar()
  : parse.cpp
  , parse.h
- INETSIZE
  : sticky.h
- INIT
  : dish.h
- INITIAL\_BUFSIZE
  : output.cpp
- INP\_PROT
  : sticky.h
- isLeft()
  : hull.cpp

### - k -

- KEYBUFSIZE
  : x11graph.cpp

### - l -

- LineType
  : x11graph.h

### - m -

- MakeDir()
  : output.h
- max
  : conrec.cpp
- MAXCELLS
  : sticky.h
- MAXHIST
  : sticky.h
- MAXNEIGH
  : sticky.h
- MAXSEED
  : sticky.h
- MAXTYPE
  : sticky.h
- MBIG
  : random.h
- MEDIUM
  : sticky.h
- MEMORYCHECK
  : warning.h
- MemoryWarning()
  : crash.cpp
  , crash.h
- MESS\_BUF\_SIZE
  : output.h
- min
  : conrec.cpp
- MOTION
  : x11graph.h
- MSEED
  : random.h
- MZ
  : random.h

### - n -

- NETSIZE
  : sticky.h
- NH\_TH
  : sticky.h
- NHHIST
  : sticky.h
- NiceMessage()
  : crash.cpp
  , crash.h
- NOPVM
  : crash.cpp
  , x11graph.cpp
- NULL\_BEAST
  : sticky.h

### - o -

- OK
  : misc.h
  , sticky.h
- OK\_BEAST
  : sticky.h
- OpenFileAndCheckExistance()
  : output.cpp
  , output.h
- OpenGZippedWriteFile()
  : output.h
- OpenReadFile()
  : output.h
  , output.cpp
- OpenWriteFile()
  : output.cpp
  , output.h
- operator<<()
  : parameter.h
  , parameter.cpp
- OUTFILE
  : x11graph.h

### - p -

- par
  : ca.cpp
  , cell.cpp
  , cell.h
  , dish.cpp
  , info.cpp
  , parameter.cpp
  , pde.cpp
  , x11graph.cpp
- ParsePar()
  : parse.cpp
  , parse.h
- PCO
  : sticky.h
- PLOTPERIODFREQUENCY
  : sticky.h
- PMUT
  : sticky.h
- PMUT2
  : sticky.h
- POTPROT
  : sticky.h

### - q -

- Quiet
  : warning.cpp

### - r -

- RANDOM()
  : random.cpp
  , random.h
  , sticky.h
- Randomize()
  : random.cpp
  , random.h
- RandomNumber()
  : random.cpp
  , random.h
- ReadDouble()
  : misc.cpp
  , misc.h
- ReadLine()
  : output.h
  , output.cpp
- ReadNumber()
  : misc.h
  , misc.cpp
- RED
  : sticky.h
- REMARK
  : sticky.h
  , misc.h
- RESIZE
  : x11graph.h

### - s -

- sat()
  : ca.cpp
- sbool()
  : parameter.cpp
  , parameter.h
- SearchToken()
  : parse.cpp
  , parse.h
- Seed()
  : random.cpp
  , random.h
- sgetpar()
  : parse.cpp
  , parse.h
- SkipLine()
  : parse.cpp
  , parse.h
- SkipToken()
  : parse.h
  , parse.cpp
- SQR
  : sqr.h
- StartSIGINTHandling()
  : crash.h
  , crash.cpp
- StartSIGSEGVHandling()
  : crash.cpp
  , crash.h
- SWAP
  : x11graph.cpp

### - t -

- TESTCELLS
  : sticky.h
- TIMESTEP
  : qtgraph.h
  , x11graph.h
- TokenInLineP()
  : parse.cpp
  , parse.h
- TRUE
  : sticky.h
  , output.h
  , misc.h

### - u -

- UNIDENTIFIED
  : warning.h

### - v -

- VERBOSE
  : x11graph.h

### - w -

- warning()
  : warning.cpp
  , warning.h
- WHITE
  : sticky.h

### - x -

- XPM
  : ca.cpp
- xsect
  : conrec.cpp

### - y -

- YesNoP()
  : misc.cpp
  , misc.h
  , output.h
  , output.cpp
- ysect
  : conrec.cpp

### - z -

- ZYGFILE
  : ca.cpp
- ZYGOTE
  : ca.cpp
- ZYGXPM
  : ca.cpp


---

Generated on Thu Aug 14 2014 22:04:02 for Tissue Simulation Toolkit by  

 1.8.6
